# Incorporating Prognostic Biomarkers into Risk Assessment Models and TNM Staging for Prostate Cancer

**DOI:** 10.3390/cells9092116

**Published:** 2020-09-17

**Authors:** Ragheed Saoud, Nassib Abou Heidar, Alessia Cimadamore, Gladell P. Paner

**Affiliations:** 1 Department of Surgery (Section of Urology), University of Chicago, Chicago, IL 60637, USA; Ragheed.Saoud@uchospitals.edu; 2 Department of Surgery (Division of Urology), American University of Beirut Medical Center, Beirut 11-0236, Lebanon; na192@aub.edu.lb; 3 Section of Pathological Anatomy, Polytechnic University of the Marche Region, School of Medicine, 60126 Ancona, Italy; alessiacimadamore@gmail.com; 4 Department of Pathology, University of Chicago, Chicago, IL 60637, USA

**Keywords:** prostate cancer, biomarkers, staging, risk assessment models, predictive scores, prognosis, molecular classifier

## Abstract

In current practice, prostate cancer staging alone is not sufficient to adequately assess the patient’s prognosis and plan the management strategies. Multiple clinicopathological parameters and risk tools for prostate cancer have been developed over the past decades to better characterize the disease and provide an enhanced assessment of prognosis. Herein, we review novel prognostic biomarkers and their integration into risk assessment models for prostate cancer focusing on their capability to help avoid unnecessary imaging studies, biopsies and diagnosis of low risk prostate cancers, to help in the decision-making process between active surveillance and treatment intervention, and to predict recurrence after radical prostatectomy. There is an imperative need of reliable biomarkers to stratify prostate cancer patients that may benefit from different management approaches. The integration of biomarkers panel with risk assessment models appears to improve prostate cancer diagnosis and management. However, integration of novel genomic biomarkers in future prognostic models requires further validation in their clinical efficacy, standardization, and cost-effectiveness in routine application.

## 1. Introduction

Extent of urological cancers is now assessed universally by the tumor-node-metastasis (TNM) system which is considered the “gold standard” for staging and benchmark for prognostication [1]. Currently, the goals of modifications in TNM staging classifications are being tailored towards a more “personalized” model rather than being based solely on anatomical factors. The 8th edition of the American Joint Committee on Cancer (8th AJCC) staging system provides major evidence-based changes by incorporating clinical and pathological variables in prostate cancer to make staging more relevant for precision medicine [2,3]. The AJCC aims at building on the anatomic basis of disease while incorporating additional clinical and pathological variables including biomarkers and risk assessment models that have been rigorously studied to predict prognosis [2].

When dealing with prostate cancer, the clinical T stage might not be optimally correlated with the biological behavior of the tumor, as demonstrated previously to be an inadequate predictor of recurrence after radical prostatectomy (RP). Moreover, it is well known that clinical staging of prostate cancer is variable due to differences in imaging modalities and subjectivity of rectal examination [2,4]. Therefore, clinical stage may vary significantly from pathological stage on RP specimens.

In current practice, prostate cancer anatomical staging alone may not be sufficient to adequately assess patient prognosis as well as to plan for management strategies. Over the past decades, multiple clinicopathological parameters and risk assessment tools for prostate cancer have been developed to better discriminate the risk and provide an adequate assessment of outcome. These clinical parameters are mainly the D’Amico derived systems that include National Comprehensive Cancer Network (NCCN), American Urologic Association (AUA), and European Association of Urology (EAU) risk stratification groups; these mainly use anatomical TNM staging, Gleason grade, as well as Prostate Specific Antigen (PSA) levels. Risk stratification models proved to be well correlated with cancer specific mortality [5,6,7,8,9].

## 2. Discussion

### 2.1. 8th AJCC Changes for Prostate Cancer Staging

Adoption of clinical and pathological data is a step forward towards more individualized and personalized medicine in order to better stratify patients in terms of prognosis. For the aforementioned reasons, AJCC had already incorporated non-anatomic factors such as serum PSA and Gleason score into “prognostic stage groups” [3]. The most recent 8th AJCC version abandoned the pathological T2 subcategorization of pT2a, pT2b, and pT2c due to the lack of prognostic significance between the subcategories [9,10,11]. More importantly, the 8th AJCC endorsed statistical models to predict survival in prostate cancer. However, the only approved prognostic models by the AJCC are for metastatic castration-resistant prostate cancer that include clinical (non-anatomic) parameters such as PSA, hemoglobin and performance status [12,13]. The AJCC did not accept prognostic models in their stringent inclusion criteria for localized prostate cancer, which included endpoints of overall survival and generalizability of the models [14].

### 2.2. Molecular Biomarkers and Gene Alterations in Prostate Cancer

The traditional PSA as well as imaging and Gleason scores provide certain prostate cancer risk stratification; however, these alone may not accurately predict the patient’s prognosis [15]. This has led to the search for additional biomarkers and prostate cancer genetic alterations that when present, can help in decision making and accurately predict prognosis. Multiple somatic single gene mutations in prostate cancer have been explored. Though it is present in about 50% of prostate cancer, *TMPRSS2*-*ERG* fusion has not been consistently associated with clinical outcomes [16], while the loss of *PTEN* tumor suppressor gene was found to be of clinical prognostic value [17]. Emerging data has also linked several germline DNA mutations to prostate cancer, namely *BRCA1*, *BRCA2*, *MSH, ATM, PALB2, CHEK2,* and *MUTYH* [18]. Individuals who may have family history of prostate cancer and positive for *BRCA2* mutation have higher rates of progression/grade reclassification while on active surveillance (AS), and lower metastasis free and overall survival after primary treatment [19,20,21,22,23]. Guidelines now recommend testing germline mutations in men diagnosed with prostate cancer and any of the following: a positive family history, high risk, regional, or metastatic prostate cancer regardless of family history, Ashkenazi Jewish ancestry, and intraductal carcinoma histology [6]. Currently, there is insufficient data to incorporate genetic mutations into prognostic algorithms or predictive models as their value in the setting of localized prostate cancer is not yet clear. However, patients with germline mutations may be counselled to follow-up closely if opting for AS [22].

Biomarkers in prostate cancer provide diagnostic, prognostic or predictive information [21]. Several diagnostic biomarkers have been explored to better detect patients with clinically significant prostate cancer while decreasing the need for prostate biopsies [24]. On the other hand, prognostic biomarkers can help stratify patients in terms of disease aggressiveness and ultimately cancer prognosis. So far, no single molecular biomarker in prostate cancer has translated into clinical use and the current trend is to explore multiple gene panels or classifiers, some of which are already commercially available.

Not all available biomarkers however have been thoroughly evaluated and validated. Currently, three mRNA-based classifiers (Prolaris, Oncotype Dx, and Decipher) are useful to evaluate tissue from biopsy or RP specimens. One protein-based biomarker (Promark) based on the expression of 8 prostate cancer specific proteins in biopsy tissue, can predict the aggressiveness of prostate cancer by providing a score from 1–100 [22].

According to the American Society of Clinical Oncology (ASCO), validated prostate cancer biomarkers should be used only in particular clinical settings, when assay result along with routine clinical factors may affect management [22]. Each of the aforementioned biomarkers can independently improve the prognostic accuracy of clinical multivariable models for identifying men with biologically significant prostate cancer [25,26,27,28]. However, there is no high-quality comparative data to determine which of the markers is the most accurate prognosticator. Longer term data is needed to determine whether their utility will impact patient quality of life and prostate cancer specific outcomes [22,29,30]. They may be useful when combined with known risk stratification systems in prostate cancer. Examples include patients who are diagnosed with low risk prostate cancer according to the D’Amico classification, but either have high volume Grade Group (GG) 1, low volume GG 2, or those who have higher risk features (e.g., Germline/somatic mutations, high PSA density) associated with GG 1 disease [21]. Though such patients may not be ideal candidates for AS, prognostic information provided by biomarkers may assist the treating physician and the patient in choosing AS vs. primary treatment [24].

### 2.3. Novel Biomarkers Integrated into Risk Assessment Models for Prostate Cancer Screening and Prognostication

#### 2.3.1. Prolaris

Prolaris is a test that can be performed on either biopsy or RP tissue. It is a sum of 31 cell cycle progression (CCP) genes and 15 reference genes [31]. It may help guide decision making in patients opting for AS versus surgery/radiation. It provides a score from 1–10; the higher the score, the higher the risk of disease progression. This score should not be utilized alone to alter decision making but rather combined with the patient’s age, PSA, clinical stage, percent positive cores, Gleason score, and AUA risk category to predict 10-year prostate cancer-specific mortality risk [31,32]. In a study of 585 men with clinically localized prostate cancer, Prolaris score was shown to be an independent predictor of prostate cancer death [32]. Per NCCN guidelines, the Prolaris biopsy score is recommended for patients with very low- and low-risk disease on biopsy and a life expectancy of 10 years [6]. However, it is also useful in men with high risk features after RP. Prolaris, combined with PSA, Gleason score, and other clinicopathologic factors including known T categories (extraprostatic extension [pT3a], seminal vesicle invasion [pT3b]), and lymph node invasion (N1) can predict 10-year risk of biochemical recurrence (BCR) after surgery (Table 1) [33,34].

#### 2.3.2. 4K-score and European Randomized Study of Screening for Prostate Cancer Rotterdam Prostate Cancer Risk Calculator (ERSPC RPCRC)

**Figure 1:**
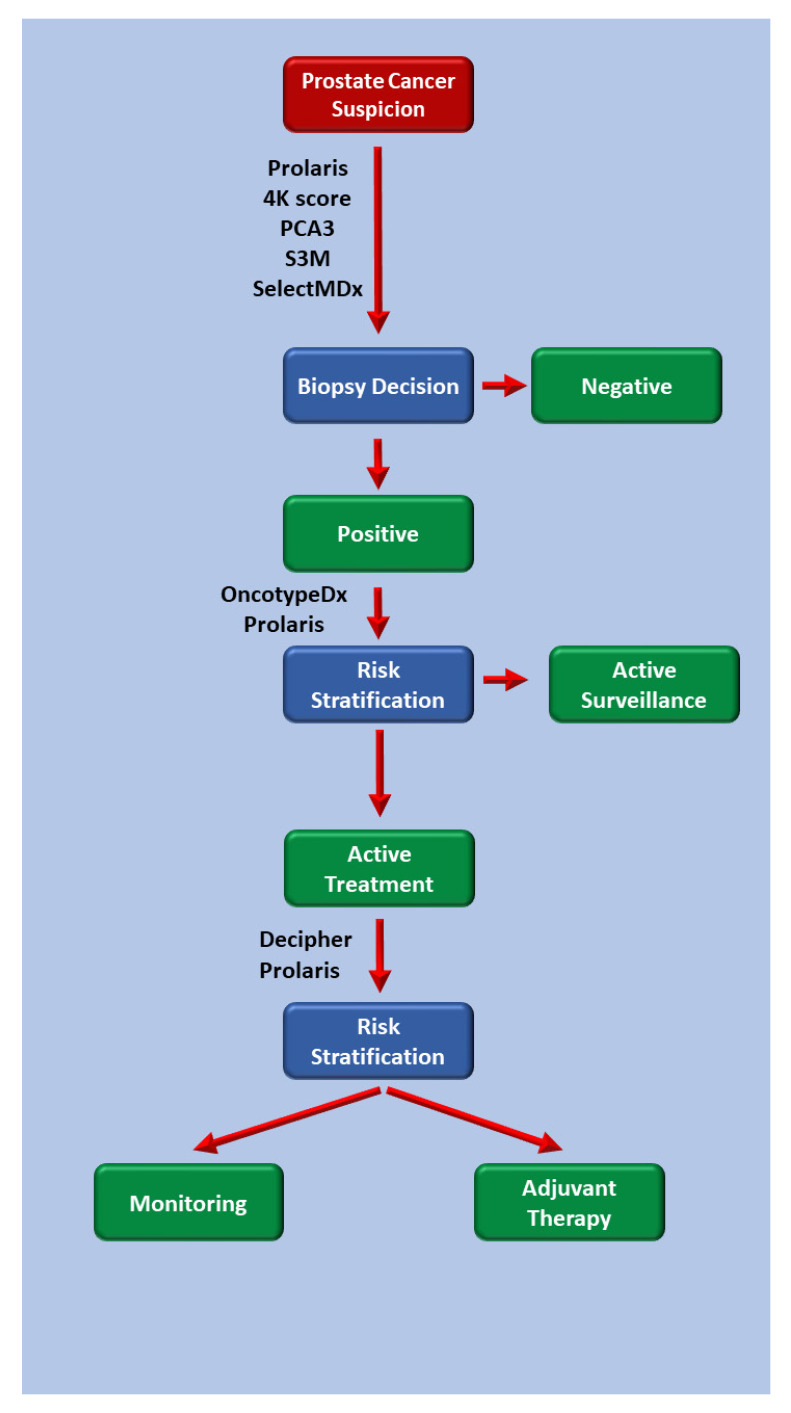
Algorithm describing the utility of prostate cancer biomarkers and risk assessment tools in clinical practice.

Numerous risk calculators (RC) are available for prostate cancer, but only six are externally validated in multiple study populations [35]. Among those, ERSPC RPCRC is the most commonly used RC as it has shown superiority in predicting men at risk for clinically significant prostate cancer [36]. However, when ERSPC RPCRC is combined with 4K score, together they are able to predict clinically significant prostate cancer more accurately than either alone and decreasing the need of unnecessary biopsies [35,37] (Figure 1). 

#### 2.3.3. PCA3

Prostate cancer antigen 3 (*PCA3*) is a gene that codes for a mRNA that is overexpressed in prostate cancer tissue. It is detectable in urine after digital rectal examination (DRE) and can be used alone to predict the presence of clinically significant prostate cancer. However, when combined with a RC, *PCA3* enhances diagnostic accuracy particularly in men undergoing initial or repeat biopsy [38]. *PCA3* based nomograms could predict initial biopsy results and avoid unnecessary biopsy in 55% of patients while missing only 2% of patients with clinically significant prostate cancer [39].

#### 2.3.4. SelectMDx

SelectMDx is a test that combines clinical parameters (PSA, DRE, prostate volume, age and family history) with two urinary prostate cancer associated genes (*HOXC6* and *DLX1*). Due to its suggested high accuracy in predicting clinically significant prostate cancer, EAU guidelines state that SelectMDx may be utilized to decide on whether an initial or repeat prostate biopsy should be performed [7]. Due to the strong predictive value of PSA/PSA density in SelectMDx, this risk assessment tool can prevent 42% of unnecessary biopsies while missing only 2% of clinically significant prostate cancer [38]. It can also exclude low risk patients from undergoing magnetic resonance imaging (MRI) prior to biopsy, as retrospective studies have shown a positive association between SelectMDx scores and (Prostate Imaging Reporting and Data System) PIRADS scores [40]. If MRI and biopsy are performed only when the SelectMDx score reflects a risk of clinically significant prostate cancer >10%, this will reduce 35% of unnecessary biopsies, avoid the detection of 52% of low risk prostate cancer and only miss 2% of clinically significant prostate cancer [36].

### 2.4. What Strategies Can Avoid Unnecessary MRI, Biopsies, and Diagnosis of Low Risk Prostate Cancer?

The best way to avoid unnecessary MRI, biopsies and diagnosis of low risk prostate cancer is to perform risk stratification of patients using a biomarker or a RC prior to pursuing unnecessary workup. When ERSPC RPCRC is performed in men with an initial negative biopsy but high suspicion of harboring cancer, the RC is able to avoid 51% of MRI’s, 69% of unnecessary repeat biopsies and 25% of low risk prostate cancer, while only missing 10% of clinically significant prostate cancer [41]. Moreover, in men who have already undergone diagnostic MRI, incorporating age and PIRADS score into the ERSPC RC can avoid one third of unnecessary biopsies after MRI (AUC = 0.84) (Table 1) [42].

S3M is a risk model created using data from the Stockholm-3 study. It combines 232 genetic polymorphisms with protein biomarkers (PSA, fPSA, iPSA etc.) and clinical variables (age, DRE, family history etc.) When used alone for screening, S3M performed significantly better than PSA for detection of clinically significant prostate cancer (AUC 0.74 versus 0.56) [43]. However it has proven especially advantageous in the context of patient selection to undergo MRI followed by targeted prostate biopsy. In a cohort of 532 men referred for prostate cancer workup, selecting only those with a risk of clinically significant prostate cancer higher than 10% by S3M could reduce unnecessary MRI and biopsies by 38%, decrease diagnosis of low risk prostate cancer by 42% while missing only 8% of clinically significant prostate cancer [43].

### 2.5. Biomarkers to Guide Decision for Active Surveillance 

Oncotype Dx Genomic Prostate Score (GPS) is performed on prostate biopsy sample that provides a risk prediction of adverse pathology on RP, which may guide physicians to choose active surveillance or pursue treatment. This 17-gene assay was combined with the CAPRA (Cancer of the Prostate Risk Assessment) score to provide a superior predictor of high-grade and high-stage disease at the time of prostatectomy [29].

Similarly, Prolaris is a 46-gene panel that can be performed on RP or biopsy specimens and helps in decision making between active surveillance and treatment [44]. Cooperberg et al. validated that the incorporation of Prolaris with CAPRA score has superior prognostic risk stratification for patients with localized prostate cancer [33]. 

### 2.6. Biomarkers to Predict Recurrence after Radical Prostatectomy

Biochemical recurrence and metastasis after RP are important measurable outcomes that have an implication on prognosis. Classical adverse pathologic features such as extraprostatic extension (pT3a), seminal vesicle involvement (pT3b), and positive surgical margins have been used to stratify patients into high risk of biochemical recurrence. Treating these patients with adjuvant radiation has been implicated with improved biochemical-free survival [45,46,47] and overall survival [48]. However, many practices offer watchful waiting for adverse pathologic features due to the indolent disease process and to decrease the risk of unwarranted radiation toxicities, especially that many trials have shown that early salvage radiation is equivalent to adjuvant radiation therapy [49]. 

Therefore, an accurate risk assessment model is needed, and not solely based on pathologic parameters, to better manage patients after RP. Genomic testing, such as the Decipher RP test, have been found to better stratify post-RP patients and identify the patients who need adjuvant radiation therapy and those who can afford to go for watchful waiting [50,51]. 

Many clinical nomograms and risk scores have been developed to predict biochemical recurrence after RP, such as the Stephenson nomogram and CAPRA-S [52,53,54]. These risk calculators rely only on clinical and pathologic information; however, if genomic information could be added to individualize stratification, this would yield more accurate information about prognosis of the disease. Indeed, Spratt et al., [55] has found that Decipher testing independently adds prognostic benefit over routine clinicopathologic variables to predict metastasis. Moreover, Spratt et al. has developed an internally validated integrated clinical-genomic risk group stratification for localized prostate cancer that outperforms traditional NCCN risk classification [56]. In addition, prospective trials confer that genomic testing post RP confers both decreased patient-related anxiety and improved decision making [57,58]. Subsequently, a defined role of genomic testing after RP has been included in the most recent NCCN guidelines [6]. However, the routine use of these genomic biomarkers and their integration in TNM/risk assessment models is still modest because of the lack of extensive cost-effectiveness studies and long-term prospective data on clinical utility.

### 2.7. Future Potential Perspectives

Since its first edition in 1968 [59], the TNM staging system has experienced regular updates and integrations, although preserving the same predominantly-anatomic based structure. With the application of new genomic technologies, the assessment of risk of cancer progression and development of metastatic diseases has improved. A modified staging system has been proposed recently by Yang et al. In addition to the traditional TNM, the authors proposed to integrate the results of the liquid biopsy designed as “B” (representing blood), as “B0” for absence and “B1” for presence of detectable circulating tumor DNA (ctDNA) [60]. So far, there are no available data on integration of ctDNA and/or circulating tumor cells with TNM or risk assessment models in prostate cancer. Moreover, the potential theranostic utility of circulating tumor cells (CTC) in the context of localized prostate cancer is still debatable and the sensitivity and specificity of technical approaches used for isolation and characterization of CTC need to be improved and standardized [61].

## 3. Conclusions

With the vast advancements in the field of personalized medicine, there is a crucial need for reliable prostate cancer prognostic biomarkers to identify patients that may benefit from different management strategies. Integration of biomarkers into risk assessment models appears to enhance prostate cancer stratification for both diagnosis and management. However, to be integrated in future prognostic and predictive models, genomic biomarkers need greater standardization to improve generalizability, validation of their clinical efficacy, and cost-effectiveness in routine application.

## Figures and Tables

**Table 1:** Available biomarkers and risk assessment tools to guide prostate cancer treatment and decision making.

Test	Type of Tissue	Genes/Biomarkers Encoded	Tool in Risk Assessment	Utility	Result
Prolaris	Biopsy	31 CCP + 15 reference genes	Combined with age, PSA, clinical stage, % positive cores, Gleason score, AUA risk category	Decision making: Active surveillance vs. Treatment	Higher score implies higher risk of cancer progression/independent predictor of prostate cancer death.
	Radical Prostatectomy		Combined with PSA, Gleason score, pathologic features of surgical specimen.	Prognostication/Need for adjuvant therapy	Predicts 10-year risk of BCR after radical prostatectomy
4-K Score	Blood	4 biomarkers: free PSA, total PSA, intact PSA, and human glandular kallikrein 2 (hk2)	Combined with ERSPC RPCRC risk calculator	Screening	Predicts presence of clinically significant prostate cancer
PCA3	Urine after DRE	Prostate Cancer Antigen 3	Combined with PSA, DRE, and risk calculator	Screening	Predicts presence of clinically significant prostate cancer: (a) On initial biopsy (b) Avoids unnecessary re-biopsy in patients with an initial negative biopsy.
Select MDx	Urine after DRE	HOXC6 and DLX1 genes	Combined with MRI, PSA, DRE, prostate volume, age, family history	Screening	Predicts presence of clinically significant prostate cancer: (a) Avoids detection of low risk prostate cancer (b) Avoids unnecessary re-biopsy
Stockholm-3 Model (S3M)		232 genetic polymorphisms + protein biomarkers (fPSA, iPSA)	Combined with age, DRE	Screening + patient selection: which patients deserve MRI +/− Biopsy.	Predicts presence of clinically significant prostate cancer (a) Avoids detection of low risk prostate cancer (b) Avoids unnecessary MRI +/- Biopsy
Oncotype Dx	Prostate biopsy	17 gene assay	Combined with CAPRA score	Decision making: Active surveillance vs. Treatment	Predicts high risk (stage & grade) disease upon eventual radical prostatectomy
